# Understanding Intracellular Transport Impairments in Maternal Protein Restriction: A Scoping Review of DOHaD-Driven Cellular Biology Perspective

**DOI:** 10.1007/s12013-026-02024-0

**Published:** 2026-03-04

**Authors:** Matheus Naia Fioretto, Isabelle Tenori Ribeiro, Maria Clara Caruso Corso, Sofia Francisco Tellaroli, Carla Beatriz Correa de Souza, Wellerson Rodrigo Scarano, Luis Antonio Justulin

**Affiliations:** Department of Structural and Functional Biology, Institute of Biosciences, São Paulo State University, Botucatu, SP Brazil

**Keywords:** DOHaD, Endocytosis, Endoplasmic reticulum, Exocytosis, Maternal malnutrition, Transport, Vesicles

## Abstract

Malnutrition remains a major global socioeconomic challenge. When it occurs during critical developmental windows (pregnancy and lactation), it can impair maternal health and predispose offspring to long-term diseases, a central concept within the Developmental Origins of Health and Disease (DOHaD). Maternal Protein Restriction (MPR) is a widely used experimental model in this field and is known to disrupt multiple organs and systems in the offspring, increasing susceptibility to metabolic disorders. However, the cellular pathways underlying these outcomes remain insufficiently explored, particularly vesicular transport and the endomembrane system. This review examined the effects of MPR on organelles related to vesicular dynamics. Following predefined inclusion and exclusion criteria, 20 PubMed articles were selected, addressing MPR-induced alterations in the cytoskeleton, endoplasmic reticulum (ER), Golgi, endocytosis, and exocytosis. Overall, evidence indicates that MPR disrupts cytoskeletal integrity—affecting actin networks in renal, adipose, muscle, and neural cells. MPR compromises ER and Golgi activities in several organs. In the placenta, endocytic activity was consistently elevated (macropinocytosis). Exocytosis was also perturbed, contributing to lipid accumulation and inflammation. Systemically, MPR altered circulating amino acid levels, impaired hematological parameters, reduced leukocyte migration, and enhanced placental amino acid transport. These findings demonstrate that MPR destabilizes cytoskeletal architecture, ER–Golgi function, and vesicular transport across multiple offspring tissues, identifying the endomembrane system as a critical target of developmental programming. These alterations suggest persistent vulnerabilities in cellular homeostasis. Therefore, we propose the term maternal diet–dependent cellular-vesicular disorders (MDCVD), establishing a conceptual framework for future mechanistic and translational studies.

**Supplementary Information:**

The online version contains supplementary material available at 10.1007/s12013-026-02024-0.

## Introduction

Scientific evidence proves that maternal exposure during pregnancy and/or lactation to adversities such as malnutrition can harm not only the mother’s health but also increase the risk of disease development in the offspring [1–3]. This proposition is investigated and supported by epidemiological and experimental data, based on a recently well-established area of ​​knowledge: the Developmental Origins of Health and Disease (DOHaD) [4–6].

As an interesting model with evident translational applicability to aspects of food insecurity and hunger, Maternal Protein Restriction (MPR) is well-documented through experimental studies that demonstrate a series of negative consequences on offspring throughout development: Risk of neurocognitive disorders [7, 8]; alterations in catabolic (corticosterone) and anabolic (insulin) hormones and basal metabolism [9–12]; impacts on the gastrointestinal system, impairing lipid metabolism and hepatic antioxidant activity [13], as well as effects on the epithelial barrier [14, 15]; structural, antioxidant, and inflammatory impacts on the heart ([16][17]) and lungs [18], increasing the risk of cardiorespiratory diseases; decreased number of nephrons, impacting renal function in osmolarity regulation and increasing the risk of hypertension [19–21]; in addition to numerous reproductive effects in females [22, 23] and males [24, 25], also being related to the developmental origin of prostate carcinoma [26, 27].

Given this overall perspective, studies within the DOHaD framework increasingly aim to elucidate global *omics* patterns and the specific metabolic–epigenetic mechanisms established during critical developmental windows, to identify biomarkers of disease susceptibility and build stronger foundations for translational research. One particularly plausible yet still underexplored mechanism involves intracellular transport—mediated by the synergistic activity of the endomembrane system (including the endoplasmic reticulum (ER), vesicles, lysosomes, and Golgi complex) through vesicular transport, endocytosis, and exocytosis. These processes are essential for intracellular communication and homeostasis, as well as for the regulation of interactions with the extracellular environment.

Constitutive endocytic and exocytic pathways maintain the dynamic equilibrium of cellular surface flow through tightly coordinated regulation [28]. Enhanced endocytosis and exocytosis rates modulate cell volume and membrane tension—mechanisms that contribute to cellular protection [29]. Conversely, dysregulation of these processes, including impaired trafficking between the ER and Golgi complex, has been associated with cellular dysfunction and various diseases [30]. Moreover, lipid-binding sensors, exchangers, and transporters play critical roles in these pathways, acting in concert with protein-mediated mechanisms whose imbalance has been linked to disorders such as atherosclerosis, dyslipidemia, diabetes, congenital lipoid adrenal hyperplasia, cancer, and male infertility [31]. The Golgi apparatus serves as the central hub of the secretory pathway, where proteins and lipids are processed and modified, and its proper structure and function depend on numerous regulatory proteins—including golgins, kinases, phosphatases, E3 ubiquitin ligases, and deubiquitinases. Disruptions in this organelle have been implicated in cancer and neurodegenerative diseases [32]. Taken together, these findings emphasize the multifaceted biological mechanisms associated with the endomembrane system, underscoring its relevance both in maintaining cellular health and in the pathogenesis of disease.

Therefore, this critical-interpretative scoping review aimed to investigate the effects of maternal protein restriction (MPR) on the endomembrane system, examining studies that effectively addressed aspects of alterations in the ER, Golgi complex, cytoskeleton, vesicles, and associated mechanisms of vesicular transport, endocytosis, and exocytosis. This objective is effectively based on three central questions: (1) What is the organ- and cell-specific mechanism of altered transport in the face of exposure to MPR?; (2) Is it possible to understand the effects of MPR in the placenta, fetuses, and different organs in the postnatal life with different transport mechanisms or organelle alterations?; (3) What are the gaps in the literature and future perspectives within the scope of the objective - MPR-Consequences in offspring-Endomembrane System and vesicular transport?

## Methods

### General Protocol

This exploratory critical-interpretative review was conducted according to the PRISMA-ScR (Preferred Reporting Items for Systematic Reviews and Meta-Analyses Extension for Scoping Reviews) guidelines [33]. The review protocol was structured according to the PRISMA-ScR recommendations (Supplementary Table 1).

### Search Strategy

A comprehensive literature search was conducted exclusively in PubMed (https://pubmed.ncbi.nlm.nih.gov/), including only studies published in English. The search was last updated on October 27, 2025. The following search terms were applied in different combinations using the Boolean operator “AND”: “*Maternal low protein diet and cytoskeleton*”; “*Maternal protein restriction and cytoskeleton*”; “*Maternal protein restriction and endoplasmic reticulum*”; “*Maternal low protein diet and endoplasmic reticulum*”; “*Maternal low protein diet and Golgi*”; “*Maternal protein restriction and Golgi*”; “*Maternal protein restriction and endocytosis*”; “*Maternal protein restriction and exocytosis*”; “*Maternal protein restriction and vesicular transport*”; “*Maternal low protein diet and vesicle*”; “*Maternal protein restriction and vesicle*”; “*Maternal low protein diet and lysosome*”; “*Maternal protein restriction and lysosome*”; “*Maternal low protein diet and endosome*”; “*Maternal protein restriction and endosome*”.

The non-use of the Boolean operator “OR” was due to the central objective of this review, which observes the direct correlation between MPR and effects on different organs and mechanisms associated with the endomembrane system and vesicular transport.

### Eligibility and Study Selection Criteria

No time restrictions were applied. All manuscript records found during the selection process were exported and stored in Microsoft Excel tables, organized according to each type of search. Eligible studies were those that: (1) evaluated the direct impact of MPR before, during gestation, and/or lactation on different organs/cells of the maternal-fetal interface (placenta) or the offspring during postnatal life, and (2) directly demonstrated alterations in components of the endomembrane system (ER, Golgi apparatus, vesicles, endosomes, and lysosomes), as well as in vesicular transport processes and their associated regulatory machinery, including endocytosis, exocytosis, and cytoskeletal dynamics. We also consider in vitro studies that mimetizated an undernourished environment, such as MPR and specific effects on the biological mechanisms supracitated.

Exclusion criteria included studies that: (1) did not establish a maternal nutritional association or effectively restricted the macromolecular proteins, (2) investigated organelles or mechanisms associated with the endomembrane system without maternal influence, or (3) did not assess the consequences of this correlation in the offspring. Of the articles excluded, we identified 9 as duplicates, 97 as not specifically using MPR, 1 preprint, 2 articles without full access, and 2 articles that did not demonstrate effects on organelles of the endomembrane system or specifically on organelles associated with these functions.

**Fig. 1:**
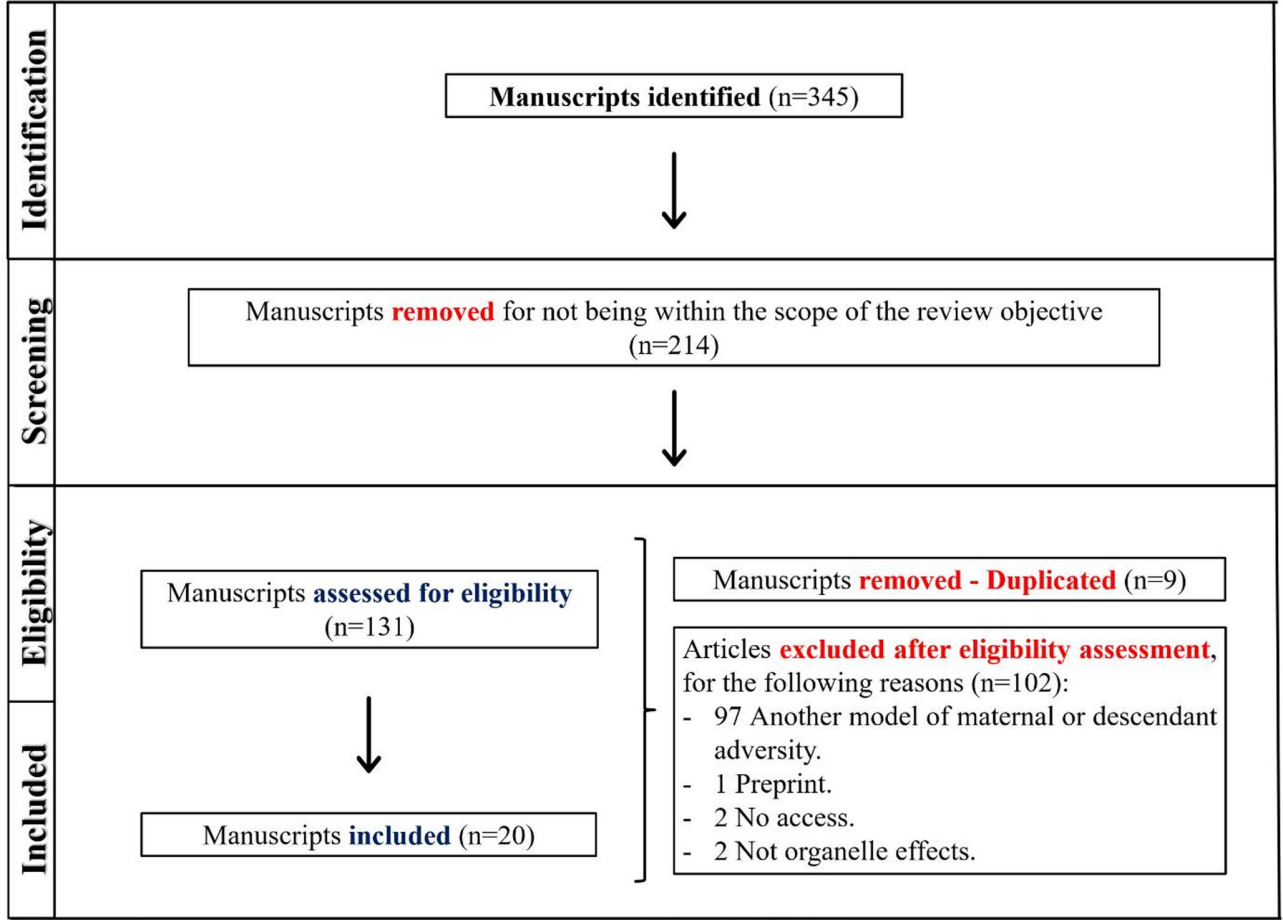
Workflow outlining the criteria for identifying, screening, determining eligibility, and including manuscripts for this scoping review

Meta-analyses, preprints, retractions, review articles (exploratory and systematic reviews), scientific integrity assessments, non-peer-reviewed reports, and comparative studies were also excluded. After the eligibility of the studies, duplicates were removed, and manuscripts that did not effectively corroborate the central scope of the review were also excluded, for reasons specified in the workflow (Fig. 1).

### Data Extraction and Analysis

Data extraction and organization were initially performed by MNF, and subsequently by all authors. The extracted information included the main results, type of protein restriction, exposure period, study model, and author details. All data were compiled in standardized spreadsheets (Microsoft Excel) to ensure consistency and transparency, as well as the individual evaluation of each manuscript by the authors.

The search strategy was intentionally restrictive to ensure conceptual specificity and mechanistic consistency, rather than exhaustive coverage. After independent screening, the inclusion or exclusion of each study was discussed collectively by all authors and is compiled in Fig. 1. Discrepancies were resolved through discussion and consensus. Due to the limited number of studies available on this specific topic, a formal risk of bias analysis was not performed.

## Results and Discussion

During the search and filtering criteria, we found 345 articles. After the screening listing the objective of the study, 214 articles were excluded because they were out of our central scope. Subsequently, we analyzed 131 manuscripts, and removed 9 (duplicated) and 102 for the following reasons: 97 Another model of maternal or descendant adversity, 1 preprint, 2 no access, and 2 not organelle effects. Therefore, we included 20 articles, 6 associated with the cytoskeleton [34–39], 6 associated with the ER and Golgi ( [27, 40–42] [43, 44]), and 8 associated with the endocytosis or exocytosis ([45] [46–52]). The main results, exposition, macromolecules, and percentage of diet, period, model, and organ are listed in Tables 1 and 2, and Table 3. Within this entire selection, we noted that only experimental articles were selected, highlighting a scientific gap regarding translational and epidemiological studies.

**Table 1:** The direct effects of MPR on the cytoskeleton in the offspring

Main results	Exposition, Macromolecules and percentage	Period	Model	Authors
Possible effects on the cell structure and neurofilaments in the offspring early in life.	Low-Protein diet (7%).	From the 5th day of gestation and through lactation.	Rats.	[37]
Decreased renal cytoskeleton homeostasis in intrauterine life.	Low-Protein diet (6% or 9%).	Gestation (embryonic period E0 until either E13.0 or E15.0).	Wistar rats.	[39]
Disorganization of myofibrils and the Z line, in addition to a reduction in the area of ​​the neuromuscular junction in the soleus muscle of offspring at 365 days of age.	Low-Protein diet (6%).	Gestation and Lactation.		[34]
Changes in actin cytoskeletal dynamics in adipose tissue of rats at 90 days.	Protein-free diet.	Lactation (first 10 days)		[35]
Upregulation of proteins related to cell cycle regulation and cytoskeletal remodeling (ER60 precursor, Thioredoxin-like 2, ARP3, G(i) alpha-1, Alpha-fetoprotein, and Tubulin alpha-1 chain in the kidneys at 16 weeks.	Low-Protein diet (9%) and/or iron deficiency.	Gestation	Wistar Rowett and Hooded Lister	[38]
Decreased transcriptional responses of cytoskeletal signaling (ACTN1, ACTN2, ACTR3, ARPC5, F2R, FN1, MYH4, MYH8, MYLK, PAK2, PPP1CB) mainly in the muscle and less in the liver of porcine progeny in early and adult life.	Low-Protein diet (6.5%) or high protein diet.	94 days post-conception and 1-, 28-, and 188-days post-partum from offspring.	German Landrace gilts	[36]

**Table 2:** The direct effects of MPR on the ER and Golgi in the offspring

Main results	Exposition, Macromolecules and percentage	Period	Model	Authors
Reduced sarcoplasmic reticulum reuptake function (imbalance in SERCA-2a, phosphorylated PLB-Ser16/total, and the sodium-calcium exchanger) in the heart (papillary muscles) of male adult rats (3 months).	Low-Protein diet (9%).	Mating period, pregnancy, and lactation.	Wistar rats.	[41]
Increased phosphorylation of hepatic eukaryotic initiation factor 2α in male rats during adulthood.	Low-Protein diet (8%).	Gestation or/and Lactation.		[42]
Females showed reduced HMG-CoA reductase, elevated e-CHOL levels, increased GCK, and decreased peIF-2α, XBP-1s, and p62. Both sexes exhibited higher PEPCK-1 and lower CHOP levels. In males, e-CHOL levels were reduced, and antioxidant enzymes (SOD-2 and catalase) were decreased in the liver.		Lactation.		[40],
Identification of potential molecular pathways (associated with ER and Golgi stress) involved in prostate carcinogenesis in offspring exposed to maternal malnutrition.	Low-Protein diet (6%).	Gestation and lactation.	Sprague Dawley rats.	[27]
Swollen Golgi membrane systems - Possible dynamisms and neuronal synthesis impacts in the male in early and adult life.				[43]
Increased mRNA expression of the ER stress marker XBP1s in the intestine (Colon) of male offspring during early and adult life.	Low-protein diet (8%). Supplemented or no Gln (1 g/kg/day) in drinking water and *PBA* (400 mg/kg/day) by gavage.			[44]

### Cytoskeletal Consequences: Potential Mechanisms Underlying Transport Dysregulation

The mechanisms by which MPR disrupts cytoskeletal organization are tightly coupled to alterations in intracellular transport, particularly via effects on microtubules and their associated motor proteins, dynein and kinesin. In parallel, actin filament impairment can compromise cellular contraction, architecture, and motility, whereas disruptions in intermediate filaments may adversely affect cell proliferation and adhesion. Together, these alterations can directly or indirectly impact vesicular and organelle transport, highlighting cytoskeletal integrity as a critical determinant of cellular homeostasis under MPR conditions. Stewart et al. [37] found that the brains of rat pups whose mothers were exposed to MPR showed structural modifications, with less cellular cytoplasm and thinner myelin sheath walls, indicating reduced dendritic development. The changes were more pronounced in the basal ganglia, hypothalamic nuclei, cerebellum, and nuclei with close connections to the cerebellum – highlighting possible aspects associated with neurofilaments.

**Table 3:** The direct effects of MPR on the vesicular transport, endocytosis, and exocytosis in the offspring

Main results	Exposition, Macromolecules and percentage	Period	Model	Authors
Syncytiotrophoblast adaptations in response to nutritional stress (in vitro amino acid depletion) with activation of the macropinocytosis machinery through repression of mammalian target of rapamycin (mTOR) signaling / Increased hCGβ and Syncytin2.	Amino acid deficiency analysis - cells maintained in a medium composed of a 7:1 mixture of DMEM with depletion of the amino acids glutamine, lysine, and arginine.	In vitro.	Human placental basal-plate samples, PHT cultures, and FSK-induced fused BeWo trophoblast cells.	[45]
Effects on the hematopoietic site of the red bone marrow - decreased migration of neutrophils and leukocytes. Systemically, there was an increase in O₂ and NO production, basal iNOS expression, and constitutive activation of NF-κB in neutrophils. High circulating levels of TNF-α and increased TNF-α mRNA expression in the spleen and liver.	Protein-free diet during the first 10 days of lactation.	Lactation.	Wistar rats.	[50]
Increase nutrient uptake through the trophoblast in the embryo. This process is triggered by amino acid reduction and regulated by the RhoA protein.	Low-Protein diet (9%).	Preimplantation Gestation.	MF1 mice.	[51]
Higher birth weight, persistent hypertension, and anxiety-like behavior. This involves early embryonic compensatory mechanisms, including increased endocytosis and LRP2 expression in the yolk sac, raising the risk of cardiovascular and metabolic diseases in adult offspring.		Preimplantation Gestation.		[52]
Decreased placental capacity to transport (Na⁺-dependent transport, system A (neutral amino acids), and diminished cationic amino acid uptake). Anionic amino acid transport via the X(–)AG system (EAAC1) is also decreased. MPR lowers steady-state mRNA levels of EAAC1 and CAT1.	Low-Protein diet (4,6%).	Gestation.	Sprague Dawley rats.	[47]
Deregulation in the concentration of hepatic amino acids (arginine, histidine, alanine, methionine, phenylalanine, and tryptophan in the mother. Alterations in hepatic capacity to transport and metabolize amino acids and fatty acids.	Low-Protein diet (6%).		Wistar rats.	[48]
Impaired antifungal immunity—reduced dissemination, phagocytosis, and control of C. albicans—alongside decreased hemoglobin, hematocrit, and MCV, with increased MCHC.	Low-Protein diet (9,5%).	Lactation (12 first days).		[49]
Increased endocytosis and lysosomal biogenesis in the trophoblast. Alterations in vesicles, megalin receptor, and cathepsin B, transcription factor TFEB - trofoblasts blastocists blastocyst trophectoderm.	Amino acid depletion and increased endocytosis and lysosomal biogenesis in the trophoblast branched-chain amino acids (BCAAs).	In vitro.	Trophoblastic cells – in vitro.	[46]

Welham et al. [39] elucidated that rats exposed to MPR during early gestation presented a decreased number of glomeruli in the embryonic period, leading to changes in gene expression in the metanephros, a reduction in prox-1 and cofilin-1, key regulators of nephrogenesis and cytoskeletal organization, leading to increased mesenchymal apoptosis and a reduction in the number of precursor cells. This study elucidates important impacts on key molecules of renal morphogenesis (prox-1 and cofilin-1), which will certainly impact organ differentiation, epithelial-mesenchymal transition, cell migration, and differentiation, potentially programming a kidney that is less responsive to long-term physiological adjustments of osmolarity. Still from the kidney response, but associated with long-term effects, MPR and/or iron restriction reduced the number of nephrons in adult male Wistar and Rowett Hooded Lister rats. Providing microarray, proteomic, and metabolic pathway analyses, the authors demonstrated impacts on cell cycle regulation (G1/S and G2/M control) and cytoskeleton remodeling (primarily of biomolecules associated with actins and tubulins) [38]). These two studies support the effects of metabolic-epigenetic programming during early development and its long-term consequences, mediated by alterations in cytoskeletal response that impair the initiation of morphogenesis. These mechanisms appear to play a fundamental role in cell cycle control disorders and long-term cell death responses; that is, aspects of dysregulation that may predispose to cellular and tissue disorders in adulthood and aging, increasing the risk of developing pathophysiologies such as hypertension or even chronic kidney disease.

Confortim et al. [34] evaluated the exposure of rats to MPR and the effects at 365 days of age on muscle fibers and neuromuscular junctions of the soleus muscle, observing structural effects on type I, IIa and IIb fibers, specifically with a decrease in collagen and disorganization of myofibrils and the Z line, in addition to a reduction in the area of ​​the neuromuscular junction. More specifically about muscle and cytoskeleton homeostasis, German Landrace sows exposed to MPR demonstrated transcriptional impacts related to tissue remodeling, cell cycle regulation, decreased actin cytoskeletal signaling (e.g., ACTN1, ACTN2, ACTR3, ARPC5, F2R, FN1, MYH4, MYH8, MYLK, PAK2, PPP1CB), and mitochondrial function in skeletal muscle and liver of offspring at different points in postnatal developmental biology. The authors argue that mitotic control and cytoskeletal changes may be important in triggering later events in muscle following MPR [36]. These two studies reinforce the effects of MPR on, primarily, essential components of muscle actin filaments at different stages of developmental biology, which affects the capacity for contraction. Furthermore, it is quite plausible to hypothesize that other mechanisms are also altered, such as energy storage capacity and muscle response, as well as the organization of the cytoskeleton, not only for contraction, but also for intracellular and organellar organization and dynamism.

**Fig. 2:**
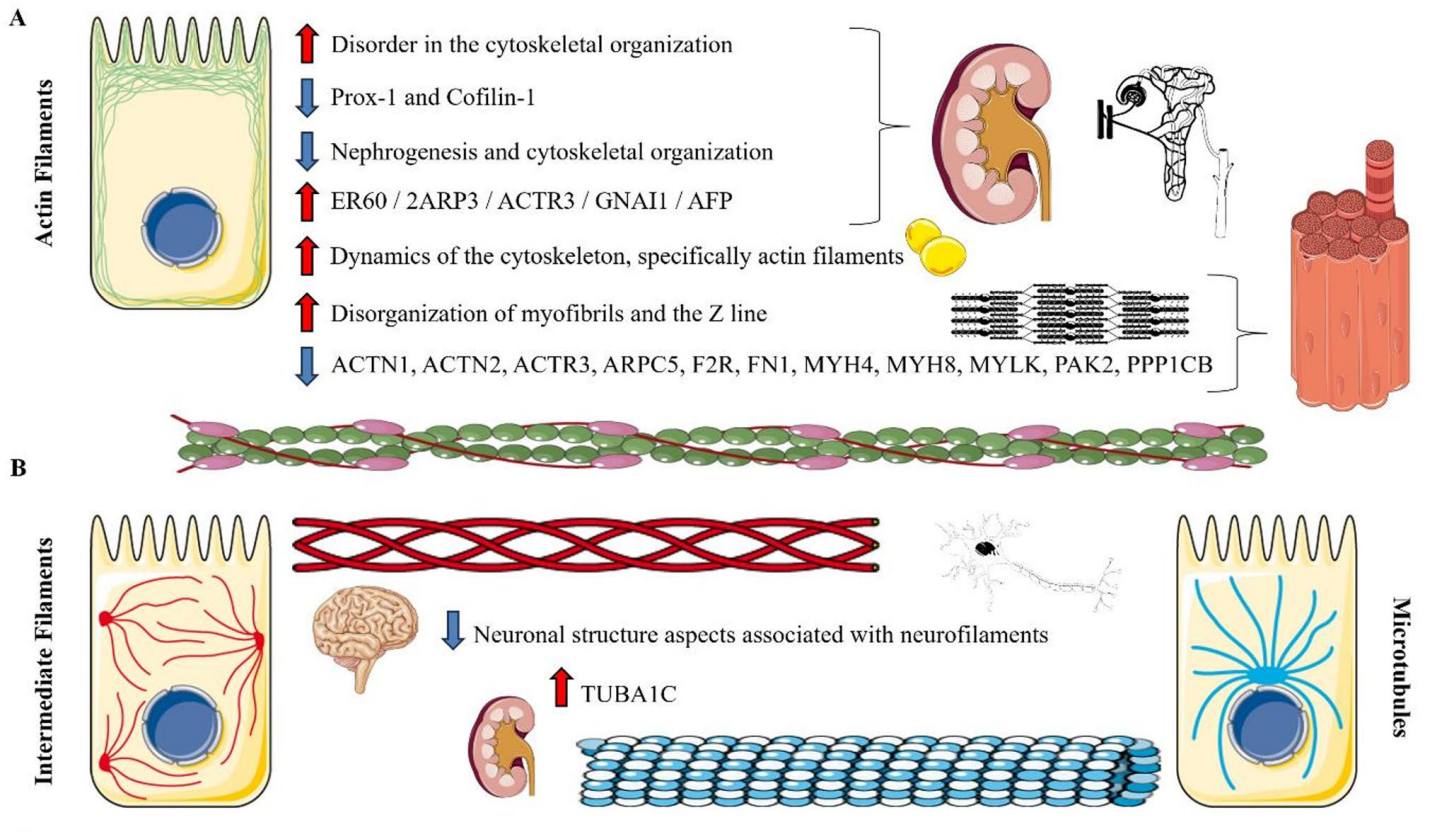
Cytoskeletal alterations induced by maternal protein restriction (MPR) in offspring. Schematic representation of the main components of the cytoskeleton and the consequences of MPR, including actin filaments (A), intermediate filaments, and microtubules (B). Actin disorganization induced by MPR can impair nephrogenesis, muscle contractile dynamics, and adipocyte function. It is suggested that alterations in intermediate filaments and microtubules disrupt intracellular organization and vesicular transport, particularly through neurofilament-associated trafficking in neural cells and microtubule-dependent processes in renal cells. Given the evidence, we hypothesize that MPR can affect the dynamics of different cytoskeletal filaments in different cells, affecting the capacity for migration, development, and transport associated with motor proteins. Adapted from Servier Medical Art (https://smart.servier.com), licensed under CC BY 4.0 (https://creativecommons.org/licenses/by/4.0/).

Evaluating adipocytes of the epididymal of adult rats exposed to a maternal protein-free diet during the first 10 days of lactation, Garcia-Souza et al. [35] observed that after insulin stimulation, there was reduced tyrosine phosphorylation of IR and IRS-1, and increased basal phosphorylation of IRS-2, Akt, and mTOR in adipocytes, demonstrating impaired adaptive effects during the insulinemic response. These changes were accompanied by an increase in GLUT4 content in the plasma membrane and alterations in the dynamics of the cytoskeleton, specifically actin filaments, as observed by fluorescence microscopy. This study opens up an important line of reasoning regarding how possible alterations in neuroendocrine and anabolic responses can directly influence the intracellular response, particularly concerning hormonal receptors, signaling pathways, and the action of the cytoskeleton in this response-sensitive cellular reorganization. Table 1; Fig. 2 summarize the effects of MPR on the offspring, focusing on the cytoskeleton disposition and function.

### Associated Deregulation by MPR from Macromolecule Destination on the Golgi and Endoplasmic Reticulum

Understanding the effects of MPR on ER function is particularly relevant given the organelle’s central roles in protein and lipid homeostasis. The rough ER is essential for protein translation, folding, post-translational modifications, and proper trafficking, while the smooth ER is critical for lipid synthesis, membrane remodeling, and detoxification processes. From an omics perspective (proteomics), rats exposed to gestational and lactational MPR showed altered levels of proteins associated with growth factors, estrogen signaling, detoxification, and energy metabolism in the prostate during early life and aging. Associated with this, these animals presented an increased incidence of prostate cancer, which was linked to the enrichment of both immediate and long-term oncological pathways. This study highlighted upregulation of pathways associated with the ER and downregulation of cytoskeletal regulation by Rho GTPase, focusing not only on hormone-dependent aspects but also on specific intracellular mechanisms associated with the ER [27], as a possible organelle associated with the developmental origins of tumors in males, specifically the prostate.

In adult male rats born to mothers exposed to MPR during gestation, cardiac functional assessment revealed increased blood pressure, prolonged time to peak contraction, and relaxation. Functional analyses demonstrated reduced post-pause force potentiation, indicating impaired sarcoplasmic reticulum calcium reuptake. These findings were supported by molecular data showing decreased myocardial SERCA-2a expression, an increased phosphorylated PLB-Ser16/total PLB ratio, and enhanced sodium–calcium exchanger activity [41]. In summary, this study establishes a direct effect of MPR on the ER and on the calcium storage and transport capacity for cardiomyocyte contraction and relaxation, based on changes in myocardial mechanics and ion transporters.

Considering aspects related to ER function, Bertasso et al. [40] evaluated the effects of MPR during lactation, observing that male and female offspring presented reduced lipids in hepatocytes adjacent to the vasculature, low levels of esterified cholesterol, and lower content of mitochondrial redox balance markers in males, in addition to altered ER stress response and estrogen receptor alpha in both sexes. Specifically, in the ER, there was lower content of C/EBP homologous protein (CHOP) in males and females, lower phosphorylated eukaryotic initiation factor-2α (peIF-2α) and X-box element-binding protein-1 (XBP-1s) in males, and lower sequestome-1 (associated with macroautophagy) in females. Interestingly, despite finding no pathological changes, the authors observed that MPR can alter the ER’s response in the medium term in a sex-specific manner, possibly as an adaptive mechanism that may impact the overall ability to metabolize lipids, carbohydrates, and amino acids.

Another experimental study elucidated that MPR decreased mRNA expression of the epithelial barrier proteins MUC2 and occludin in the offspring’s colon in early life and adulthood, an aspect associated with increased mRNA expression of the ER stress marker XBP1s, colonic permeability, inflammation, and decreased goblet cells. Interestingly, the authors found that maternal supplementation (4-phenylbutyrate and glutamine) during gestation and lactation partially prevented the increased expression of ER stress markers in adulthood, parameters reversed by maternal supplementation [44].

Rats exposed to MPR during gestation and lactation exhibited, in adulthood, increased hepatic phosphorylation of eIF2α (Ser51) concomitant with reduced phosphorylation of Akt1 (Ser473) [42]. This condition represents a key regulatory event in the integrated stress response, typically triggered by ER stress and nutrient deprivation, which selectively favors stress-adaptive transcripts, suggesting impaired translation, post-translational aspects, and protein activity.

**Fig. 3:**
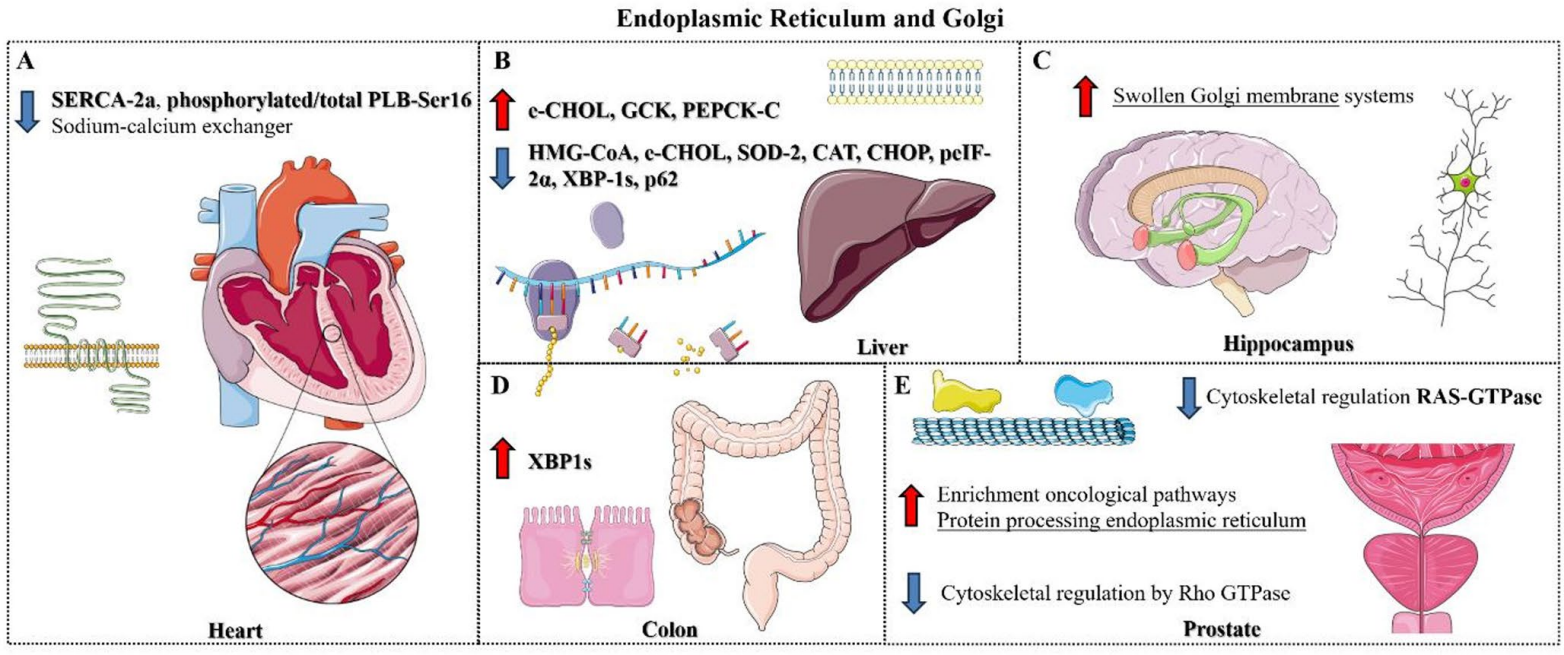
Smooth and rough endoplasmic reticulum (ER) and Golgi apparatus alterations induced by maternal protein restriction (MPR) in offspring. Altered mechanisms associated with cardiac transport and contraction response (A), lipid and amino acid metabolism, and hepatic antioxidant action (B), neuronal alterations (C), as well as intestinal lipid transport (D). In the prostate, the effects on ER stress appear to be associated with the origins of prostate cancer development, while the consequences in neural cells are potentially associated with post-translational modifications (E). Given the altered biomarkers and pathways, it is possible to infer a potential risk of MPR affecting these organelles directly associated with the development of different cells, as well as impacting synthetic capacity and vesicular transport. Adapted from Servier Medical Art (https://smart.servier.com), licensed under CC BY 4.0 (https://creativecommons.org/licenses/by/4.0/).

Specifically, regarding ultrastructural aspects, Castro-Chavira et al. [43] evaluated the impact of MPR on hippocampal pyramidal neurons in young offspring over 22 weeks, observing significant cellular changes in dynamics, including abnormal mitochondrial density, swollen Golgi membrane systems, and increased lipofuscin and multivesicular bodies. This study elucidates, albeit only from an ultrastructural perspective and not a molecular one, the possible organelle-specific effects of MPR on neurons, which may impact synthesis capacity and behaviors associated with short- and long-term memory. Table 3; Fig. 3 summarize the effects of MPR on the offspring, focusing on the ER and Golgi disposition and function.

### Specific Effects on the Vesicular Transport and Endomembrane System

The syncytiotrophoblast exhibits a remarkable ability to adapt to nutritional stress by activating alternative nutrient uptake mechanisms through macropinocytosis, a process induced by the suppression of the mammalian target of rapamycin (mTOR) signaling pathway. During periods of amino acid scarcity, mTOR inhibition enhances macropinocytic activity, enabling the syncytiotrophoblast to sustain the uptake of essential nutrients. Studies in pregnant mice have demonstrated that mTOR inhibition stimulates macropinocytosis in the syncytiotrophoblast; however, when this process is blocked, the phenotypes associated with fetal growth restriction are exacerbated. Thus, the ability of the syncytiotrophoblast to undergo macropinocytosis represents a critical cellular adaptation essential for maintaining fetal survival and growth under nutrient-deprived conditions [45].

Silva et al. [50] demonstrated that the offspring subjected to maternal malnutrition during lactation exhibited inhibited neutrophil migration and impaired recruitment of leukocytes from the bone marrow to the circulation. In addition, circulating neutrophils from these animals showed reduced phagocytic activity, along with increased production of O₂ and NO, higher basal iNOS expression, and constitutive activation of the transcription factor NF-κB. Elevated serum levels of TNF-α and increased TNF-α mRNA expression in the spleen and liver were also observed. These findings indicate that maternal malnutrition during lactation can interfere with the innate immune response in adulthood, leading to long-lasting alterations in neutrophil activation and regulation. Specifically, although this study does not directly address alterations in endocytic or exocytic mechanisms, the reported effects on bone marrow function suggest potential disruptions in vesicular transport processes involved in hematopoiesis. Likewise, the observed impairments in neutrophil signaling may reflect direct disturbances in exocytosis-dependent pathways.

Sun et al. [51] demonstrated that blastocysts from mothers subjected to MPR showed increased endocytic activity in the TE, characterized by a higher number and volume of vesicles per cell, including those containing endocytosed ligands and fluids, as well as lysosomes. An increase in the expression of megalin (Lrp2), a receptor from the low-density lipoprotein (LDL) family, was also observed. In an in vitro model replicating the reduced amino acid composition of uterine fluid, decreased levels of leucine, isoleucine, and valine stimulated endocytosis in the TE, mediated by RhoA GTPase signaling. These findings indicate that embryos exposed to MPR during the preimplantation period enhance endocytic activity in extra-embryonic lineages as a compensatory mechanism in response to nutrient limitation.

Other studies have shown that MPR limited to the preimplantation period can lead to increased birth weight, persistent hypertension in adulthood, and anxiety-related behavioral abnormalities, particularly in females. Moreover, these effects persisted even after embryo transfer, indicating that they originate from early embryonic responses to MPR. The visceral endoderm of the yolk sac was identified as a mediator of this adaptation, enhancing nutrient uptake and transport. The expression of the LRP2 protein, involved in the endocytosis of maternal proteins, also increased even when MPR occurred only at the beginning of gestation. Thus, MPR activates embryonic plasticity mechanisms to sustain growth, but these early adjustments may result in excessive growth and a higher risk of cardiovascular and metabolic diseases in adulthood [52].

Malandro et al. [47] investigated the effect of MPR on amino acid transport across the placenta in rats and observed that the amino acid ratios in maternal-fetal serum were reduced, suggesting a reduction in nutrient transfer to the fetus. The authors observed that Na+-dependent neutral amino acid transport mediated by the A system and cationic amino acid uptake were reduced in apical and basal trophoblastic membrane vesicles, in addition to decreased Na+-dependent anionic amino acid uptake by the X (-) AG system (EAAC1) in the basal membrane. Another important finding was that MPR led to a decrease in steady-state mRNA content for EAAC1 and CAT1 (y+ system), which suggests that the reduction in the synthesis of transporter proteins is responsible for the decrease in transport activity.

Another very interesting study demonstrated that first-time mothers exposed to MPR during pregnancy exhibited increased micro- and macrovesicles of fat, necrosis, and inflammation in their liver during gestation, with an increase in the total concentration of hepatic amino acids at the beginning of gestation and a decrease at the end of gestation in the dams, specifically in relation to arginine, histidine, alanine, methionine, phenylalanine, and tryptophan. These results certainly indicate hepatic impacts on amino acid biosynthesis and catabolism, as well as on fat metabolism [48], effects that may be associated with problems in the rough ER and vesicular transport.

Using an in vitro model of amino acid depletion, Caetano et al. [46] evaluated how trophoblastic cells respond through endocytosis and lysosomal biogenesis. Isoleucine (ILE) deficiency emerged as the strongest inducer of these processes, markedly increasing the formation of vesicles enriched in megalin and cathepsin B, with this response detectable as early as the blastocyst stage. In addition, TFEB was identified as a key regulator of this histotrophic adaptation, demonstrating its translocation from the cytoplasm to the nucleus under ILE deficiency and during mTORC1 inhibition. The study also showed that depletion of specific amino acids promotes lysosomal biogenesis and nuclear TFEB activation.

**Fig. 4:**
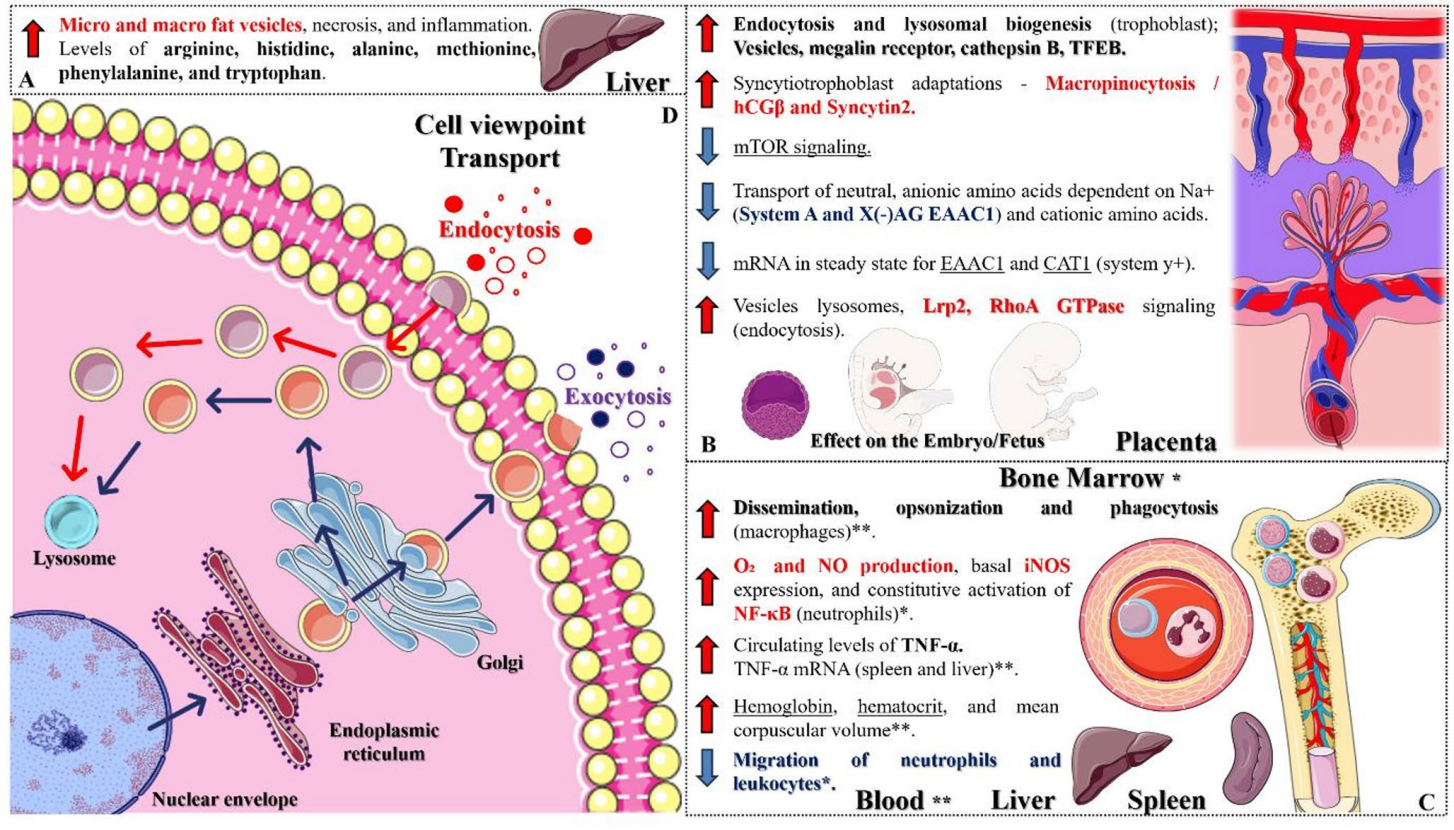
Mechanisms of vesicular transport, endocytosis, and exocytosis affected by Maternal Protein Restriction (MPR). Specific effects on hepatic micro- and macrovesicles (A), embryo-fetal development, transporters and placental endocytic capacity (B), as well as on bone marrow maturation and hematopoietic activity, with systemic effects (C) that may be correlated to a priori problems in the regulatory mechanisms and effectiveness of vesicular transport and the endomembrane system, as well as in aspects of endocytosis and exocytosis (D). From the results, it is plausible to consider that different cells are affected by MPR in terms of vesicular transport, which may lead to negative effects on the capacity for internalization of biomolecules, as well as on the capacity for release. Adapted from Servier Medical Art (https://smart.servier.com), licensed under CC BY 4.0 (https://creativecommons.org/licenses/by/4.0/).

At 80 days of age, rats exposed to MPR during lactation exhibited impaired dissemination and phagocytosis of opsonized yeasts, and they were unable to block germ tube formation or eliminate C. albicans to the same extent as the control group. The diet produced a reduction in hematological parameters (hemoglobin, packed cell volume, mean corpuscular volume) and increased mean corpuscular hemoglobin concentration [49]. Based on the results of this study, it is possible to hypothesize that the capacity for cellular interaction and phagocytosis is altered in these models, which may be associated with systemic disorders that could be related to problems in hematopoiesis and cell maturation. Table 2; Fig. 4 summarize the effects of MPR on the offspring, focusing on vesicular transport, endocytosis, and exocytosis.

### General Insights and Interpretation

Specifically, MPR varies in severity and induces a wide range of organ-specific alterations that depend on the timing of exposure (preconception, gestation, and/or lactation). These effects may be further exacerbated by adverse postnatal environments. Such dietary adversity disrupts embryonic and fetal development by impairing placental function and affecting neural, renal, and hepatic development, altering muscle physiology, and disturbing systemic processes involving circulating cells and adipose tissue. Collectively, these changes increase susceptibility to chronic non-communicable diseases, including cardiovascular and intestinal disorders and prostate cancer. Although this review captures only a fraction of the known consequences of MPR, the compiled evidence consistently highlights cellular responses involving cytoskeletal organization, the ER, the Golgi apparatus, and vesicular trafficking.

Overall, the findings of this review reveal compelling insights into cellular dynamics and adaptive responses to MPR. MPR affects multiple organelles and essential processes that sustain cellular and systemic homeostasis. We highlight a set of alterations that directly or indirectly disrupt vesicle-mediated transport, providing a strong foundation for future investigations into these mechanisms within both cellular biology and the DOHaD framework.

From a holistic and biological perspective, we often describe cytoskeletal, ER, and general transport aspects as interconnected; however, it is important to emphasize that some mechanisms are combined, and others are isolated, within organelle-specific functions. Here, based on the analyzed studies, we highlight how possible alterations in the cytoskeletal framework or function, in association with or without motor proteins, can have a direct or indirect correlation with the function of the ER, Golgi apparatus, and vesicular transport. Furthermore, it is important to emphasize that these mentioned alterations do not necessarily have a direct effect on, or only on, vesicular transport, clarifying the enormous magnitude of the processes by which filaments, vesicles, and organelles participate within cellular dynamism. Finally, from a critical-interpretative point of view, the biological insights provided are important for mechanistic interpretation and possible further observations in future analyses to understand, in ever greater depth, the correlation between MPR and effects on the endomembrane system, in the sense of specific organelles or mechanisms adjacent to their transport functions.

Another highly relevant aspect is the close association between alterations in signaling pathways and processes linked to the endomembrane system across critical temporal windows—embryo–fetal development, early postnatal life, adulthood, and aging—as well as the malnutrition exposure during pre-gestational, gestational, and/or lactational periods. These time-dependent insults can program offspring phenotypes through nuclear, transcriptional, and phenotypic changes mediated by epigenetic mechanisms. Environmental stressors are capable of reprogramming the germline epigenome, thereby transmitting disease susceptibility across generations via transgenerational epigenetic inheritance [53]. Specifically, epigenetic modifications (DNA methylation, histone modifications, and non-coding RNAs) are central mediators of how early-life environments shape long-term health outcomes and form the mechanistic basis of the DOHaD framework [54]. These processes contribute to increased susceptibility to obesity, fatty liver disease, hypertension, and type 2 diabetes. Accordingly, epigenetic regulation reflects adaptive responses in human bioenergetics, placental development, metabolic homeostasis, and environmental exposures that collectively promote chronic disease risk in adulthood [1]. To better elucidate these mechanisms, placental epigenomics represents a particularly promising strategy, integrating advanced approaches such as multiomics analyses, non-coding RNA profiling, mixture-exposure modeling, and longitudinal assessment of health outcomes beyond early childhood [55]. Notably, the effects compiled in this review converge on the potential association of epigenetic regulation of cellular processes involving the cytoskeleton, ER, and Golgi apparatus. However, the epigenetic underpinnings of MPR and their links to endomembrane dysfunction remain insufficiently explored. In this context, the present review highlights critical knowledge gaps and underscores the need for integrative studies capable of linking nutritional adversity, epigenetic programming, and endomembrane system and vesicular transport across the life course.

#### Cytoskeleton

Examining cytoskeletal alterations is crucial for understanding cellular structure and intracellular dynamics, given the central role of actin filaments in maintaining cortical structure, cellular architecture, and movement, and the essential function of microtubules—together with the motor proteins dynein and kinesin—in driving vesicular and organelle transport, in addition to mitotic and division processes. Here, we demonstrate that MPR can affect actin filaments in renal cells, adipocytes, and skeletal striated muscle fibers, while in relation to microtubules, the effect is on neural and renal cells. The main results suggest disorganization in the filaments, which certainly negatively impacts intracellular transport and metabolism. From a molecular point of view, we can establish the following correlations: Direct effects on renal branching morphogenesis - aspects of cell division (AFP, Prox-1 and cofilin-1), on differentiation and morphogenetic movements (2ARP3, ACTR3, TUBA1C), post-translational processes (ER60), and intracellular signaling (GNAI1). Regarding the kidneys, the literature demonstrates that MPR reduces renal development during fetal life, mediated by hypoxia factors (HIF-1α signaling pathway) [56] and by the dysregulation of ciliogenesis and ciliary elongation factors in the renal tubular epithelium, inhibition of β-catenin signaling, and induction of cell apoptosis [21]. Furthermore, MPR alters renal tubular transporters, which are regulated by physiological mechanisms, and is a risk factor for the development of hypertension [19]. Although these findings are not directly linked to vesicular transport, they indicate that cytoskeletal disturbances during embryonic and fetal development disrupt intracellular organization, which may underlie long-term risk. Such alterations are likely to affect not only endocytosis and exocytosis, but also passive and active membrane transport, as well as transporter function.

In skeletal striated muscle fibers, the impacts on sarcomere arrangement and muscle contraction mechanisms (ACTN1, ACTN2, ACTR3, MYH4, MYH8, MYLK), cell signaling and second messenger response (F2R, PAK2, PPP1CB), as well as extracellular impacts on the matrix (FN1) and cytoskeleton polymerization and depolymerization (ARPC5), are evident, which can be directly associated with the biosynthetic capacity and intracellular organization of these cells. Experimental evidence indicates that MPR induces skeletal muscle and neural dysfunctions characterized by denervation, demyelination of the sciatic nerve, increased centralized nuclei, and upregulation of atrogenes, consistent with muscle atrophy [57]. Additional studies show that MPR disrupts oxidative muscle fibers, particularly in the soleus, affecting glycolytic flux, the Krebs cycle, and the electron transport chain, alongside anabolic, myogenic, and structural pathways [58, 59]. These alterations likely reflect disturbances in cytoskeletal integrity and intracellular organization. Moreover, MPR impairs insulin signaling in skeletal muscle, evidenced by reduced Akt2 and GLUT4 expression, and induces mitochondrial dysfunction through altered fusion (Mfn1/2), fission (Fis1), and biogenesis pathways (Pgc1β, Nrf1, Esrra) [60, 61]. In neural tissue, alterations in neurofilament-related processes suggest impaired anterograde and retrograde vesicular transport, potentially compromising neurotransmitter synthesis, trafficking, and reuptake.

#### ER-Golgi Function

Regarding the rough and smooth ER, as well as the Golgi apparatus, we identified disturbances across cardiac, ininal, hepatic, cerebral, and prostatic tissues. In the heart, these alterations were closely linked to impaired calcium handling, potentially driven by disrupted ER calcium uptake—reflected in changes to SERCA-2a, the sodium–calcium exchanger, and phosphorylated/total PLB-Ser16—ultimately compromising myocardial contractility. The literature demonstrates that MPR decreases the number of cardiomyocytes and connexin 43 expression, leads to cardiomyocyte degeneration, increases fibrotic aspects, and iNOS [62]. Furthermore, Harvey et al. [63] demonstrated that maternal nutritional restriction leads to a decrease in the protein content of troponin I and ryanodine in adult male rats, impacting excitation-contraction coupling. These data are consistent with the aspects observed in the analyzed studies, representing negative adaptations that may be associated with alterations in the ER. In the liver, the results were associated with systemic alterations correlated with lipid and carbohydrate metabolism (CHOL, GCK, PEPCK-C), as well as a decrease in cholesterol biosynthesis capacity (HMG-CoA), antioxidant capacity (SOD-2, CAT) and autophagy (p62), in addition to impacts on ER stress and post-transcriptional and post-translational aspects (CHOP, peIF-2α, XBP-1s) in the intestine. Other studies with MPR have elucidated that this condition suppresses fatty acyl-CoA reductase 1 (FAR1) and leads to hepatic ferroptosis, aspects that impact the autophagic response [64]. Another study also revealed that MPR impacts AMPK signaling and hepatic autophagy [65]. Liu et al. [66] demonstrated that rats exposed to MPR showed decreased hepatic molecules of the response to misfolded proteins (UPR), heat shock protein 4 L (Hspa4l), mitogen-activated protein kinase 10 (Mapk10), and ER-to-nucleus signaling 2 (Ern2).

Collectively, these studies align with disruptions of the ER and Golgi complex. In neural cells, a prominent finding is Golgi membrane swelling, suggesting impaired protein processing and trafficking. Early malnutrition alters fetal brain tryptophan–serotonin–kynurenine metabolism, while in adulthood it leads to elevated levels of these metabolites in the brainstem, cortex, and hippocampus [67]. Such changes may reflect compromised neurotransmitter synthesis and post-translational processing linked to Golgi dysfunction. In the prostate, early-life nutritional adversity is associated with increased prostate carcinoma incidence, dysregulation of oncogenic pathways, and heightened ER stress responses during development and aging. Together, these findings support a functional ER–cytoskeleton–Golgi axis as a potential mechanism underlying developmental programming of disease, including cancer susceptibility, and highlight the need to investigate these organelles across additional tissues and life stages.

#### Vesicular Transport

In relation to endocytic processes, the analyzed studies consistently demonstrated enhanced endocytosis and trophoblastic lysosomal biogenesis, characterized by increased vesicle formation, upregulation of the megalin receptor, cathepsin B, and TFEB activation. Additionally, syncytiotrophoblast adaptations promoted macropinocytosis—evidenced by elevated hCGβ and syncytin-2 and reduced mTOR—and further increased lysosomal activity through Lrp2 and RhoA GTPase signaling. The placenta is a fundamental site for understanding the maternal-fetal interface, and ultrastructural and functional alterations in this temporary organ certainly play a key role in fetal development and in aspects of metabolic/fetal programming associated with the origins of susceptibility to disease development in offspring. Rosário et al. [68] elucidated that, in microvillous plasma membranes isolated from placentas of animals with MPR, the protein expression of sodium-coupled neutral amino acid transporter (SNAT)2 and large neutral amino acid transporters 1 and 2 was reduced on days 19 and 21 of gestation, leading to inhibition of placental insulin, mTOR, and STAT3 signaling, which is associated with a downregulation of placental amino acid transporters. MPR decreases the junctional zone of the placenta and the expression of Pcdh12, a marker of glycogenic trophoblastic cells (Gonzalez et al., 69). Reed et al. [70] elucidated that sheep exposed to malnutrition showed decreased CXCL12 and VEGF, and increased TNFα, in addition to increased expression of IGF-II mRNA and decreased IGFBP-3. It is quite plausible to consider that the endocytic alterations observed in the placenta are specific effects of the organelle, dependent on altered paracrine and neuroendocrine signaling, bringing an important perspective to placental cell biology in response to systemic changes in the face of dietary challenges.

In the bone marrow and blood circulation, we observed an increased macrophage response through dissemination, opsonization, and phagocytosis, O₂ and NO production, basal iNOS expression, and constitutive NF-κB (neutrophil) activation. These results certainly explain the altered adaptive mechanisms in the hematopoietic site of the offspring, which may indicate cellular ultrastructural alterations associated with impaired innate and adaptive immune response. Based on these results, the literature also elucidates that the maturation of natural killer cells is linked to maternal MHC and not fetal MHC, so that mothers with MHC deficiency produce fetuses with greater growth restriction, demonstrating direct impacts on the bone marrow [71]. Protein depletion, therefore, may be a direct effect of hematopoietic cell disorder in the fetus, leading to short- to long-term consequences on immune capacity. Gong et al. [72] elucidated that rats exposed to maternal caloric restriction showed, from the isolation of mesenchymal stem cells from bone marrow (BMSCs), increased proliferation and an adipogenic molecular profile (PPARγ, miR-30d, miR-103, PPARγ, C/EPBα, ADRP, LPL, SREBP1) and negative regulation of Wnt signaling (LRP5, LEF-1, β-catenin, ZNF521, and RUNX2). In a comparative study, Oreffo et al. [73] observed the impact of MPR on the differentiation of mesenchymal stem cells in offspring, which indicates that perhaps not only the differentiation capacity of the bone marrow is altered, but also that of the embryonic germ layers during critical periods of development, probably mediated by the capacity of organelles involved in cellular transport.

In the vesicular transport and potential exocytosis mechanisms, we observed an increase in micro and macro fat vesicles, necrosis, and inflammation. Levels of arginine, histidine, alanine, methionine, phenylalanine, and hepatic tryptophan were elevated. Furthermore, there was an increase in circulating levels of TNF-α. An increase in TNF-α mRNA (spleen and liver), hemoglobin, hematocrit, and mean corpuscular volume was also observed. And the migration of neutrophils and leukocytes associated with the red bone marrow decreased. The data indicate increased Na⁺-dependent transport of neutral, anionic, and cationic amino acids, accompanied by elevated placental EAAC1 and CAT1 mRNA expression. Although these findings primarily reflect changes in membrane transporter–mediated uptake rather than direct effects on exocytosis, altered amino acid availability is likely to influence cellular biosynthesis and, secondarily, secretory and exocytotic pathways. In this context, exosomes—vesicles released via fusion of multivesicular bodies with the plasma membrane—serve as key mediators of intercellular communication by transporting bioactive cargo, a mechanism well described in chronic liver disease and potentially relevant to placental signaling under nutritional stress [74]. Alterations in certain key molecules, such as the guanosine triphosphatase Rac2, can impair the exocytosis of primary granules in mast cells due to changes in the cytoskeleton and other organelles of these cells [75], specifically impacting the immune response. Exosomes can mediate communication between cells, facilitating processes such as antigen presentation, in addition to the presence of small RNAs, including microRNAs, in mast cells [76] - a very interesting fact that converges aspects of the exocytic pathway and epigenetic factors.

Pregnancies with fetal growth restriction exhibit reduced placental PPARγ activity. In mice, the loss of trophoblastic PPARγ mimics impaired fetal growth and defective adipogenesis. Trophoblasts promote pre-adipocyte differentiation through the transfer of exosomal PPARγ, stimulating the expression of adipogenic genes. Therefore, the placenta serves as an important source of PPARγ, and insufficient delivery of exosomes to fetal pre-adipocytes at the end of gestation is a key mechanism leading to fetal growth restriction [77]. This study is quite interesting because it associates translational and preclinical aspects of intrauterine growth restriction (a condition that is also a consequence of MPR exposure) with problems in exocytosis and cell communication. Although a single target is highlighted, the evidence supports a multifactorial impairment of placental exocytic capacity with consequences for fetal development and long-term offspring health. Placental function is tightly regulated by hormones, soluble factors, and extracellular vesicles, which are critical in both physiological and pathological states such as preeclampsia and diabetes [78]. Notably, altered levels of total and placenta-derived exosomes in maternal and fetal circulation have been proposed as biomarkers of intrauterine growth restriction [79], and experimental studies show that maternal stress modifies small extracellular vesicle profiles and placental–fetal targeting, contributing to growth restriction [80].

#### Future Perspectives

Within the context of metabolic programming and its integration with aspects of cellular transport, we elucidate some possible associative alterations in certain hormonal pathways such as insulin, estrogen, and glucocorticoids. Specifically, peptide hormones like insulin require vesicular transport for their release, and alterations in intracellular mechanisms can lead to insufficient concentrations of these hormones. Furthermore, the hormonal response associated with membrane or intracellular receptors (such as some steroids) also depends on intracellular mechanisms and signaling pathways (e.g., PI3K/Akt), and the action of these hormones can modulate the formation, trafficking, and fusion of vesicle membranes, cytoskeleton polymerization and depolymerization, genomic responses and transcription factors associated with the ER response, mitochondria-ER interactions, and vesicular flow, among others, which ultimately can also modulate biochemical aspects of these cells such as anabolism and catabolism. Although this review has focused on intracellular mechanisms, there is certainly an inherent correlation between not only the endocrine aspect, but also the neural aspect, which expands the view beyond the cell, and also extends to understanding its biological cause/effect relationship in accordance with the systemic environment.

For future perspectives, a more mechanistic understanding of how MPR affects cellular function—particularly intracellular and vesicular transport—is urgently needed. Upcoming studies should investigate the signaling pathways that activate or suppress endocytic and exocytic processes, as well as the protein interactions required for these mechanisms (e.g., clathrin, COPI/COPII complexes, SNAREs). Although several studies describe the effects of MPR on different organs and cell types, few directly address its impact on the endomembrane system and organelles involved in intracellular trafficking. Key targets for future investigation include proteins associated with endocytosis (e.g., AP2 complex, dynamin-2, PI3K-related pathways, GTPases, caveolin-1) and exocytosis (e.g., SNAREs, Munc proteins, Rab GTPases, calcium-dependent channels, CD63, CD9, CD81), as well as mechanisms related to exosome biogenesis. It is also essential to explore how these alterations manifest not only at the cellular level but systemically, given that some disruptions may contribute to hormone-dependent disorders. To advance understanding of how MPR programs offspring phenotypes through disruptions in vesicular transport, integrative approaches combining ultrastructural and advanced imaging techniques (e.g., confocal microscopy), multiomics analyses (proteomics, transcriptomics, and epigenomics), extracellular vesicle profiling, and single-cell technologies will be essential. Together, these strategies can identify robust biomarkers and signaling pathways with translational relevance, strengthening the biological basis of DOHaD and enabling the development of both preventive and interventional strategies within precision medicine.

## Limitations

It is important to acknowledge that, although a formal risk-of-bias assessment was not performed, substantial heterogeneity exists across the included studies. Differences in MPR protocols, experimental designs, technical approaches, and biological models (in vitro and in vivo; rats, mice, and gilts) can lead to divergent outcomes, reflecting the distinct adaptive capacities of each system to nutritional challenges such as MPR and potentially limiting direct translational extrapolation. Nevertheless, by systematically synthesizing the principal findings and recurrent biological alterations involving the cytoskeleton, ER, Golgi apparatus, and vesicular transport, this review provides an integrated framework that can guide future investigations toward reduced methodological heterogeneity, improved reproducibility, and stronger translational relevance.

In addition, it is important to emphasize that even though it is a scoping review, there are still evident limitations that need to be highlighted. Given the stated objective, we can list the limitations of the lack of signaling pathways or deep biological mechanisms associated with MPR dysregulation in the cytoskeleton, ER, Golgi complex, and vesicular transport, but this is quite associated with limitations within the literature. One limitation within our strategies was the failure to investigate the general effects on active and passive transport across the membrane, an aspect that may also be associated, even if indirectly, with disturbances in vesicular transport. Another limitation was the lack of emphasis on the effects of different hormones, which certainly have impacts on hormone-dependent cellular responses. Perhaps, finally, the greatest limitation is the lack of translational and clinical data that could better support and facilitate the extrapolation of experimental data; however, this is an important point we highlight here so that future studies can improve findings regarding biomarkers, as well as provide more basis for epidemiological studies, which would certainly fill the main gap highlighted at the beginning of the manuscript. However, despite the limitations inherent in any study, this review highlights a specific organelle perspective on vesicular transport, tracing this biological mechanism as a potential factor associated with the origins of the development of cellular disorders in response to the dietary challenge of MPR.

## Conclusion

Therefore, MPR disrupts cytoskeletal organization in renal, adipose, muscular, and neural tissues; impairs endoplasmic reticulum ER and Golgi complex function—particularly in the heart; and alters placental endocytosis, as well as hepatic and bone marrow exocytotic processes. Collectively, these findings reveal organ-specific cellular vulnerabilities within the endomembrane system and vesicular transport machinery in offspring exposed to maternal malnutrition at different developmental windows. Such alterations suggest persistent functional disturbances that originate during the embryonic–fetal period and extend into postnatal life.

Based on this integrative evidence, we propose that these alterations represent developmental origins of cytoskeletal, ER, Golgi complex, and vesicular transport dysfunctions driven by maternal malnutrition, providing a unifying conceptual framework for future mechanistic and translational investigations. To describe this spectrum of diet-dependent cellular alterations, we introduce the term maternal diet-dependent cellular-vesicular disorders (MDCVD). Importantly, MDCVD is proposed as a mechanistic heuristic framework rather than a novel pathological entity or a replacement for the DOHaD paradigm. Instead, it complements DOHaD by integrating diet-dependent cellular phenotypes with a specific emphasis on the endomembrane system and vesicular transport mechanisms.

## Supplementary Information

Below is the link to the electronic supplementary material.

Supplementary Material 1

## Data Availability

No datasets were generated or analysed during the current study.

## References

[1] Hoffman, D. J., Powell, T. L., Barrett, E. S., & Hardy, D. B. (2021). Developmental origins of metabolic diseases. *Physiological Reviews*, *101*(3), 739–795. 10.1152/physrev.00002.2020 33270534 10.1152/physrev.00002.2020 PMC8526339

[2] Langley-Evans, S. C., Phillips, G. J., Benediktsson, R., Gardner, D. S., Edwards, C. R., Jackson, A. A., & Seckl, J. R. (1996). Protein intake in pregnancy, placental glucocorticoid metabolism and the programming of hypertension in the rat. *Placenta*, *17*(2–3), 169–172. 10.1016/s0143-4004(96)80010-5 8730887 10.1016/s0143-4004(96)80010-5

[3] Vithayathil, M. A., Gugusheff, J. R., Ong, Z. Y., Langley-Evans, S. C., Gibson, R. A., & Muhlhausler, B. S. (2018). Exposure to maternal cafeteria diets during the suckling period has greater effects on fat deposition and sterol regulatory element binding Protein-1c (SREBP-1c) gene expression in rodent offspring compared to exposure before birth. *Nutrition & Metabolism*, *15*, 17. 10.1186/s12986-018-0253-3 29467799 10.1186/s12986-018-0253-3 PMC5815184

[4] Barker, D. J., Osmond, C., Golding, J., Kuh, D., & Wadsworth, M. E. (1989). Growth in utero, blood pressure in childhood and adult life, and mortality from cardiovascular disease. *BMJ (Clinical Research ed)*, *298*(6673), 564–567. 10.1136/bmj.298.6673.564 2495113 10.1136/bmj.298.6673.564 PMC1835925

[5] Suzuki, K. (2018). The developing world of dohad. *Journal of Developmental Origins of Health and Disease*, *9*(3), 266–269. 10.1017/S2040174417000691 28870276 10.1017/S2040174417000691

[6] World Health Organization (2024, March 1). Malnutrition. https://www.who.int/news-room/fact-sheets/detail/malnutrition

[7] Belluscio, L. M., Berardino, B. G., Ferroni, N. M., Ceruti, J. M., & Cánepa, E. T. (2014). Early protein malnutrition negatively impacts physical growth and neurological reflexes and evokes anxiety and depressive-like behaviors. *Physiology & Behavior*, *129*, 237–254. 10.1016/j.physbeh.2014.02.051 24607933 10.1016/j.physbeh.2014.02.051

[8] Crossland, R. F., Balasa, A., Ramakrishnan, R., Mahadevan, S. K., Fiorotto, M. L., & Van den Veyver, I. B. (2017). Chronic maternal Low-Protein diet in mice affects Anxiety, Night-Time energy expenditure and sleep Patterns, but not circadian rhythm in male offspring. *PloS One*, *12*(1), e0170127. 10.1371/journal.pone.0170127 28099477 10.1371/journal.pone.0170127 PMC5242516

[9] de Oliveira, J. C., Gomes, R. M., Miranda, R. A., Barella, L. F., Malta, A., Martins, I. P., Franco, C. C., Pavanello, A., Torrezan, R., Natali, M. R., Lisboa, P. C., Mathias, P. C., & de Moura, E. G. (2016). Protein restriction during the last third of pregnancy malprograms the neuroendocrine axes to induce metabolic syndrome in adult male rat offspring. *Endocrinology*, *157*(5), 1799–1812. 10.1210/en.2015-1883 27007071 10.1210/en.2015-1883 PMC5393358

[10] Passos, M. C., da Fonte Ramos, C., Dutra, S. C., Mouço, T., & de Moura, E. G. (2002). Long-term effects of malnutrition during lactation on the thyroid function of offspring. *Hormone and Metabolic research = Hormon- Und Stoffwechselforschung = Hormones Et Metabolisme*, *34*(1), 40–43. 10.1055/s-2002-19966 11833001 10.1055/s-2002-19966

[11] Peixoto-Silva, N., Frantz, E. D., Mandarim-de-Lacerda, C. A., & Pinheiro-Mulder, A. (2011). Maternal protein restriction in mice causes adverse metabolic and hypothalamic effects in the F1 and F2 generations. *The British Journal of Nutrition*, *106*(9), 1364–1373. 10.1017/S0007114511001735 21736811 10.1017/S0007114511001735

[12] Su, Y., Jiang, X., Li, Y., Li, F., Cheng, Y., Peng, Y., Song, D., Hong, J., Ning, G., Cao, Y., & Wang, W. (2016). Maternal low protein isocaloric diet suppresses pancreatic β-Cell proliferation in mouse offspring via miR-15b. *Endocrinology*, *157*(12), 4782–4793. 10.1210/en.2016-1167 27754789 10.1210/en.2016-1167

[13] Lillycrop, K. A., Rodford, J., Garratt, E. S., Slater-Jefferies, J. L., Godfrey, K. M., Gluckman, P. D., Hanson, M. A., & Burdge, G. C. (2010). Maternal protein restriction with or without folic acid supplementation during pregnancy alters the hepatic transcriptome in adult male rats. *The British Journal of Nutrition*, *103*(12), 1711–1719. 10.1017/S0007114509993795 20211039 10.1017/S0007114509993795

[14] Adachi, H., Ishiyama, S., & Mochizuki, K. (2023). Dietary protein restriction during pregnancy and/or early weaning reduces the number of goblet cells in the small and large intestines of female mice pups. *Biochemistry and Biophysics Reports*, *34*, 101475. 10.1016/j.bbrep.2023.101475 37197734 10.1016/j.bbrep.2023.101475 PMC10183655

[15] Dos Santos, I. B. L., Fioretto, M. N., Jorge, M. S., Barata, L. A., Ribeiro, I. T., Franzolin, A. M. L., Stoppa, E. G., Mattos, R., Portela, L. M. F., Emílio Silva, M. T., Santos, D., de Arruda, S. A. A., Miranda, J. R., Lima, H., C. A., & Justulin, L. A. (2025). Maternal protein restriction impairs intestinal morphophysiology and antioxidant system in young male offspring rats. *Experimental Cell Research*, *446*(1), 114464. 10.1016/j.yexcr.2025.114464 39986598 10.1016/j.yexcr.2025.114464

[16] Barros, M. A., De Brito Alves, J. L., Nogueira, V. O., Wanderley, A. G., & Costa-Silva, J. H. (2015). Maternal low-protein diet induces changes in the cardiovascular autonomic modulation in male rat offspring. Nutrition, metabolism, and cardiovascular diseases. *NMCD*, *25*(1), 123–130. 10.1016/j.numecd.2014.07.011 25287449 10.1016/j.numecd.2014.07.011

[17] Sobrinho Lemos, L., Naia Fioretto, M., Tenori Ribeiro, I., Annibal Barata, L., Alessandra Maciel, F., Leonardo Fagundes, F., Mattos, R., Portela, F., Barboza, L. M., de Oliveira, J. M. S., Emílio, B., de Almeida, K., Dos Santos, A., Lima, S. A. H., de Arruda Miranda, C. A., Zambrano, J. R., E., & Justulin, L. A. (2025). Maternal protein restriction promotes cardiac disorders by disrupting heart developmental morphophysiology in young male offspring Rats. Experimental cell research, 114795. Advance online publication. 10.1016/j.yexcr.2025.114795 10.1016/j.yexcr.2025.114795 41106765

[18] Naia Fioretto, M., Maciel, F. A., Barata, L. A., Ribeiro, I. T., Basso, C. B. P., Ferreira, M. R., Dos Santos, S. A. A., Mattos, R., Baptista, H. S., Portela, L. M. F., Padilha, P. M., Felisbino, S. L., Scarano, W. R., Zambrano, E., & Justulin, L. A. (2024). Impact of maternal protein restriction on the proteomic landscape of male rat lungs across the lifespan. *Molecular and Cellular Endocrinology*, *592*, 112348. 10.1016/j.mce.2024.112348 39218056 10.1016/j.mce.2024.112348

[19] Mesquita, F. F., Gontijo, J. A., & Boer, P. A. (2010). Expression of renin-angiotensin system signalling compounds in maternal protein-restricted rats: Effect on renal sodium excretion and blood pressure. *Nephrology Dialysis Transplantation: Official Publication of the European Dialysis and Transplant Association - European Renal Association*, *25*(2), 380–388. 10.1093/ndt/gfp505 19793932 10.1093/ndt/gfp505

[20] Sene, L. B., Mesquita, F. F., de Moraes, L. N., Santos, D. C., Carvalho, R., Gontijo, J. A., & Boer, P. A. (2013). Involvement of renal corpuscle MicroRNA expression on epithelial-to-mesenchymal transition in maternal low protein diet in adult programmed rats. *PloS One*, *8*(8), e71310. 10.1371/journal.pone.0071310 23977013 10.1371/journal.pone.0071310 PMC3747155

[21] Wang, J., Zhou, P., Zhu, L., Guan, H., Gou, J., & Liu, X. (2023). Maternal protein deficiency alters primary cilia length in renal tubular and impairs kidney development in fetal rat. *Frontiers in Nutrition*, *10*, 1156029. 10.3389/fnut.2023.1156029 37485393 10.3389/fnut.2023.1156029 PMC10358357

[22] Côrtes, L. S., Silveira, H. S., Lupi, L. A., de Mello Santos, T., Cavariani, M. M., Domeniconi, R. F., Gaiotte, L. B., de Morais Oliveira, D. A., Justulin, L. A., & de Almeida Chuffa, L. G. (2021). Maternal protein restriction impairs nutrition and ovarian histomorphometry without changing p38MAPK and PI3K-AKT-mTOR signaling in adult rat ovaries. *Life Sciences*, *264*, 118693. 10.1016/j.lfs.2020.118693 33130082 10.1016/j.lfs.2020.118693

[23] Guzmán, C., García-Becerra, R., Aguilar-Medina, M. A., Méndez, I., Merchant-Larios, H., & Zambrano, E. (2014). Maternal protein restriction during pregnancy and/or lactation negatively affects follicular ovarian development and steroidogenesis in the prepubertal rat offspring. *Archives of Medical Research*, *45*(4), 294–300. 10.1016/j.arcmed.2014.05.005 24819035 10.1016/j.arcmed.2014.05.005

[24] Cavariani, M. M., de Mello Santos, T., Pereira, D. N., de Almeida Chuffa, L. G., Pinheiro, F., Scarano, P. F., W. R., & Domeniconi, R. F. (2019). Maternal protein restriction differentially alters the expression of AQP1, AQP9 and VEGFr-2 in the epididymis of rat offspring. *International Journal of Molecular Sciences*, *20*(3), 469. 10.3390/ijms20030469 30678254 10.3390/ijms20030469 PMC6387270

[25] Genovese, P., Herrera, E., Riaño, V., & Bielli, A. (2019). Subnutrition effects during pregnancy and lactation on mitosis, apoptosis and androgen receptor expression in the rat testis. *Reproduction in Domestic animals = Zuchthygiene*, *54*(3), 506–513. 10.1111/rda.13385 30499612 10.1111/rda.13385

[26] Santos, S. A. A., Camargo, A. C., Constantino, F. B., Colombelli, K. T., Mani, F., Rinaldi, J. C., Franco, S., Portela, L. M. F., Duran, B. O. S., Scarano, W. R., Hinton, B. T., Felisbino, S. L., & Justulin, L. A. (2019). Maternal Low-Protein diet impairs prostate growth in young rat offspring and induces prostate carcinogenesis with aging. *The Journals of Gerontology Series A Biological Sciences and Medical Sciences*, *74*(6), 751–759. 10.1093/gerona/gly118 29762647 10.1093/gerona/gly118

[27] Santos, S. A. A., Camargo, A. C. L., Constantino, F. B., Colombelli, K. T., Portela, L. M. F., Fioretto, M. N., Vieira, J. C. S., Padilha, P. M., de Oliveira, M. B., Felisbino, S. L., Carvalho, R. F., & Justulin, L. A. (2020). Identification of potential molecular pathways involved in prostate carcinogenesis in offspring exposed to maternal malnutrition. *Aging (Albany Ny)*, *12*(20), 19954–19978. 10.18632/aging.104093 33049715 10.18632/aging.104093 PMC7655221

[28] Gundelfinger, E. D., Kessels, M. M., & Qualmann, B. (2003). Temporal and Spatial coordination of exocytosis and endocytosis. *Nature Reviews Molecular Cell Biology*, *4*(2), 127–139. 10.1038/nrm1016 12563290 10.1038/nrm1016

[29] Mao, F., Yang, Y., & Jiang, H. (2021). Endocytosis and exocytosis protect cells against severe membrane tension variations. *Biophysical Journal*, *120*(24), 5521–5529. 10.1016/j.bpj.2021.11.019 34838532 10.1016/j.bpj.2021.11.019 PMC8715248

[30] Tang, V. T., & Ginsburg, D. (2023). Cargo selection in Endoplasmic reticulum-to-Golgi transport and relevant diseases. *The Journal of Clinical Investigation*, *133*(1), e163838. 10.1172/JCI163838 36594468 10.1172/JCI163838 PMC9797344

[31] Soffientini, U., & Graham, A. (2016). Intracellular cholesterol transport proteins: roles in health and disease. Clinical science (London, England:1979), 130(21), 1843–1859. 10.1042/CS20160339 10.1042/CS20160339 27660308

[32] Li, J., Ahat, E., & Wang, Y. (2019). Golgi structure and function in Health, Stress, and diseases. *Results and Problems in Cell Differentiation*, *67*, 441–485. 10.1007/978-3-030-23173-6_19 31435807 10.1007/978-3-030-23173-6_19 PMC7076563

[33] Tricco, A. C., Lillie, E., Zarin, W., O'Brien, K. K., Colquhoun, H., Levac, D., Moher, D., Peters, M. D. J., Horsley, T., Weeks, L., Hempel, S., Akl, E. A., Chang, C., McGowan, J., Stewart, L., Hartling, L., Aldcroft, A., Wilson, M. G., Garritty, C., Lewin, S., … Straus, S. E. (2018). PRISMA Extension for Scoping Reviews (PRISMA-ScR): Checklist and Explanation. Annals of internal medicine, 169(7), 467–473 10.7326/M18-0850 10.7326/M18-0850 30178033

[34] Confortim, H. D., Jerônimo, L. C., Centenaro, L. A., Pinheiro, F., Brancalhão, P. F., Matheus, R. M. M., S. M., & Torrejais, M. M. (2015). Effects of aging and maternal protein restriction on the muscle fibers morphology and neuromuscular junctions of rats after nutritional recovery. *Micron (Oxford England:1993)*, *71*, 7–13. 10.1016/j.micron.2014.12.006 25597842 10.1016/j.micron.2014.12.006

[35] Garcia-Souza, E. P., da Silva, S. V., Félix, G. B., Rodrigues, A. L., de Freitas, M. S., Moura, A. S., & Barja-Fidalgo, C. (2008). Maternal protein restriction during early lactation induces GLUT4 translocation and mTOR/Akt activation in adipocytes of adult rats. *American Journal of Physiology Endocrinology and Metabolism*, *295*(3), E626–E636. 10.1152/ajpendo.00439.2007 18559980 10.1152/ajpendo.00439.2007

[36] Oster, M., Murani, E., Metges, C. C., Ponsuksili, S., & Wimmers, K. (2014). High- and low-protein gestation diets do not provoke common transcriptional responses representing universal target pathways in muscle and liver of Porcine progeny. *Acta Physiologica (Oxford England)*, *210*(1), 202–214. 10.1111/apha.12192 24188291 10.1111/apha.12192

[37] Stewart, R. J., Merat, A., & Dickerson, J. W. (1974). Effect of a low protein diet in mother rats on the structure of the brains of the offspring. *Biology of the Neonate*, *25*(3–4), 125–134. 10.1159/000240685 4451687 10.1159/000240685

[38] Swali, A., McMullen, S., Hayes, H., Gambling, L., McArdle, H. J., & Langley-Evans, S. C. (2011). Cell cycle regulation and cytoskeletal remodelling are critical processes in the nutritional programming of embryonic development. *PloS One*, *6*(8), e23189. 10.1371/journal.pone.0023189 21858025 10.1371/journal.pone.0023189 PMC3157362

[39] Welham, S. J., Riley, P. R., Wade, A., Hubank, M., & Woolf, A. S. (2005). Maternal diet programs embryonic kidney gene expression. *Physiological Genomics*, *22*(1), 48–56. 10.1152/physiolgenomics.00167.2004 15827236 10.1152/physiolgenomics.00167.2004

[40] Bertasso, I. M., de Moura, E. G., Pietrobon, C. B., Cabral, S. S., Kluck, G. E. G., Atella, G. C., Manhães, A. C., & Lisboa, P. C. (2022). Low protein diet during lactation programs hepatic metabolism in adult male and female rats. *The Journal of Nutritional Biochemistry*, *108*, 109096. 10.1016/j.jnutbio.2022.109096 35779796 10.1016/j.jnutbio.2022.109096

[41] de Belchior, A. C., Freire, D. D. Jr, da Costa, C. P., Vassallo, D. V., Padilha, A. S., & Santos, D., L (2016). Maternal protein restriction compromises myocardial contractility in the young adult rat by changing proteins involved in calcium handling. *Journal of Applied Physiology (Bethesda Md :1985)*, *120*(3), 344–350. 10.1152/japplphysiol.00246.2015 26586904 10.1152/japplphysiol.00246.2015

[42] Sohi, G., Revesz, A., & Hardy, D. B. (2013). Nutritional mismatch in postnatal life of low birth weight rat offspring leads to increased phosphorylation of hepatic eukaryotic initiation factor 2 α in adulthood. *Metabolism: Clinical and Experimental*, *62*(10), 1367–1374. 10.1016/j.metabol.2013.05.002 23768545 10.1016/j.metabol.2013.05.002

[43] Castro-Chavira, S. A., Aguilar-Vázquez, A. R., Martínez-Chávez, Y., Palma, L., Padilla-Gómez, E., & Diaz-Cintra, S. (2016). Effects of chronic malnourishment and aging on the ultrastructure of pyramidal cells of the dorsal hippocampus. *Nutritional Neuroscience*, *19*(8), 329–336. 10.1179/1476830515Y.0000000009 25730173 10.1179/1476830515Y.0000000009

[44] Désir-Vigné, A., Haure-Mirande, V., de Coppet, P., Darmaun, D., Le Dréan, G., & Segain, J. P. (2018). Perinatal supplementation of 4-phenylbutyrate and glutamine attenuates Endoplasmic reticulum stress and improves colonic epithelial barrier function in rats born with intrauterine growth restriction. *The Journal of Nutritional Biochemistry*, *55*, 104–112. 10.1016/j.jnutbio.2017.12.007 29413485 10.1016/j.jnutbio.2017.12.007

[45] Shao, X., Cao, G., Chen, D., Liu, J., Yu, B., Liu, M., Li, Y. X., Cao, B., Sadovsky, Y., & Wang, Y. L. (2021). Placental trophoblast syncytialization potentiates macropinocytosis via mTOR signaling to adapt to reduced amino acid supply. *Proceedings of the National Academy of Sciences of the United States of America*, *118*(3), e2017092118. 10.1073/pnas.2017092118 33402432 10.1073/pnas.2017092118 PMC7826386

[46] Caetano, L., Eckert, J. J., Johnston, D., Chatelet, D. S., Tumbarello, D. A., Smyth, N. R., Ingamells, S., Price, A., & Fleming, T. P. (2021). Blastocyst trophectoderm endocytic activation, a marker of adverse developmental programming. *Reproduction (Cambridge England)*, *162*(4), 289–306. 10.1530/REP-21-0234 34338217 10.1530/REP-21-0234

[47] Malandro, M. S., Beveridge, M. J., Kilberg, M. S., & Novak, D. A. (1996). Effect of low-protein diet-induced intrauterine growth retardation on rat placental amino acid transport. *The American Journal of Physiology*, *271*(1 Pt 1), C295–C303. 10.1152/ajpcell.1996.271.1.C295 8760058 10.1152/ajpcell.1996.271.1.C295

[48] Navarro-Meza, M., Cardador-Martínez, A. B., Vazquez-Martínez, O., Cruz-Ramos, J. A., Santoyo-Telles, F., Bejarano-Carrillo, J. A., Manzano-Hernández, A. J., & Díaz-Muñoz, M. (2019). Changes in amino acid profiles and liver alterations in pregnant rats with a high carbohydrate/low protein diet. *Annals of Hepatology*, *18*(2), 345–353. 10.1016/j.aohep.2018.11.004 31060976 10.1016/j.aohep.2018.11.004

[49] Prestes-Carneiro, L. E., Laraya, R. D., Silva, P. R., Moliterno, R. A., Felipe, I., & Mathias, P. C. (2006). Long-term effect of early protein malnutrition on growth curve, hematological parameters and macrophage function of rats. *Journal of Nutritional Science and Vitaminology*, *52*(6), 414–420. 10.3177/jnsv.52.414 17330504 10.3177/jnsv.52.414

[50] Silva, S. V., Garcia-Souza, E. P., Moura, A. S., & Barja-Fidalgo, C. (2010). Maternal protein restriction during early lactation induces changes on neutrophil activation and TNF-alpha production of adult offspring. *Inflammation*, *33*(2), 65–75. 10.1007/s10753-009-9159-6 19830535 10.1007/s10753-009-9159-6

[51] Sun, C., Velazquez, M. A., Marfy-Smith, S., Sheth, B., Cox, A., Johnston, D. A., Smyth, N., & Fleming, T. P. (2014). Mouse early extra-embryonic lineages activate compensatory endocytosis in response to poor maternal nutrition. *Development (Cambridge England)*, *141*(5), 1140–1150. 10.1242/dev.103952 24504338 10.1242/dev.103952

[52] Watkins, A. J., Ursell, E., Panton, R., Papenbrock, T., Hollis, L., Cunningham, C., Wilkins, A., Perry, V. H., Sheth, B., Kwong, W. Y., Eckert, J. J., Wild, A. E., Hanson, M. A., Osmond, C., & Fleming, T. P. (2008). Adaptive responses by mouse early embryos to maternal diet protect fetal growth but predispose to adult onset disease. *Biology of Reproduction*, *78*(2), 299–306. 10.1095/biolreprod.107.064220 17989357 10.1095/biolreprod.107.064220

[53] King, S. E., & Skinner, M. K. (2020). Epigenetic transgenerational inheritance of obesity susceptibility. *Trends in Endocrinology and Metabolism: TEM*, *31*(7), 478–494. 10.1016/j.tem.2020.02.009 32521235 10.1016/j.tem.2020.02.009 PMC8260009

[54] Bianco-Miotto, T., Craig, J. M., Gasser, Y. P., van Dijk, S. J., & Ozanne, S. E. (2017). Epigenetics and dohad: From basics to birth and beyond. *Journal of Developmental Origins of Health and Disease*, *8*(5), 513–519. 10.1017/S2040174417000733 28889823 10.1017/S2040174417000733

[55] Lapehn, S., & Paquette, A. G. (2022). The placental epigenome as a molecular link between prenatal exposures and fetal health outcomes through the dohad hypothesis. *Current Environmental Health Reports*, *9*(3), 490–501. 10.1007/s40572-022-00354-8 35488174 10.1007/s40572-022-00354-8 PMC9363315

[56] Gomes, J. S., Sene, L. B., Lamana, G. L., Boer, P. A., & Gontijo, J. A. R. (2023). Impact of maternal protein restriction on Hypoxia-Inducible factor (HIF) expression in male fetal kidney development. *PloS One*, *18*(5), e0266293. 10.1371/journal.pone.0266293 37141241 10.1371/journal.pone.0266293 PMC10159110

[57] Ersoy, U., Altinpinar, A. E., Kanakis, I., Alameddine, M., Gioran, A., Chondrogianni, N., Ozanne, S. E., Peffers, M. J., Jackson, M. J., Goljanek-Whysall, K., & Vasilaki, A. (2024). Lifelong dietary protein restriction induces denervation and skeletal muscle atrophy in mice. *Free Radical Biology & Medicine*, *224*, 457–469. 10.1016/j.freeradbiomed.2024.09.005 39245354 10.1016/j.freeradbiomed.2024.09.005 PMC7617303

[58] Valente, J. S., Perez, É. S., Zanella, T., Gutierrez, B. T., de Paula, T., Alcantara Dos Santos, S. A., da Silva Duran, B. O., Carvalho, R. F., Justulin, L. A., de Almeida Fantinatti, B. E., & Dal-Pai-Silva, M. (2021). Maternal protein restriction changes structural and metabolic gene expression in the skeletal muscle of aging offspring rats. *Histology and Histopathology*, *36*(8), 853–867. 10.14670/HH-18-337 33843034 10.14670/HH-18-337

[59] Valente, J. S., Colombelli, K. T., Pereira, L. L., Perez, É. S., Zanella, T., Delgado, B. T., Fioretto, A. Q., Padovani, M. N., Vechetti, C. R., Damasceno, I. J., Justulin, D. C., L. A.,&, & Dal-Pai-Silva, M. Jr (2025). Aerobic exercise acts differentially on proteins from glucose and glycogen pathways in the SOL and PL muscles of offspring rats submitted to a low-protein maternal diet. *Biochemical and Biophysical Research Communications*, *752*, 151483. 10.1016/j.bbrc.2025.151483 39954356 10.1016/j.bbrc.2025.151483

[60] Awata, K., Shoji, H., Arai, Y., Santosa, I., Tokita, K., Murano, Y., & Shimizu, T. (2024). Maternal protein restriction inhibits insulin signaling and insulin resistance in the skeletal muscle of young adult rats. *Juntendo Iji zasshi = Juntendo Medical Journal*, *70*(2), 142–151. 10.14789/jmj.JMJ23-0029-OA 39430205 10.14789/jmj.JMJ23-0029-OA PMC11487360

[61] Vidyadharan, V. A., Betancourt, A., Smith, C., Blesson, C. S., & Yallampalli, C. (2024). Maternal Low-Protein diet leads to mitochondrial dysfunction and impaired energy metabolism in the skeletal muscle of male rats. *International Journal of Molecular Sciences*, *25*(23), 12860. 10.3390/ijms252312860 39684571 10.3390/ijms252312860 PMC11641076

[62] Amer, M. G., Mohamed, N. M., & Shaalan, A. A. M. (2017). Gestational protein restriction: Study of the probable effects on cardiac muscle structure and function in adult rats. *Histology and Histopathology*, *32*(12), 1293–1303. 10.14670/HH-11-883 28217832 10.14670/HH-11-883

[63] Harvey, T. J., Murphy, R. M., Morrison, J. L., & Posterino, G. S. (2015). Maternal nutrient restriction alters Ca2 + Handling properties and contractile function of isolated left ventricle bundles in male but not female juvenile rats. *PloS One*, *10*(9), e0138388. 10.1371/journal.pone.0138388 26406887 10.1371/journal.pone.0138388 PMC4583465

[64] Guo, Y., Zhou, P., Qiao, L., Guan, H., Gou, J., & Liu, X. (2023). Maternal protein deficiency impairs peroxisome biogenesis and leads to oxidative stress and ferroptosis in liver of fetal growth restriction offspring. *The Journal of Nutritional Biochemistry*, *121*, 109432. 10.1016/j.jnutbio.2023.109432 37657642 10.1016/j.jnutbio.2023.109432

[65] Devarajan, A., Rajasekaran, N. S., Valburg, C., Ganapathy, E., Bindra, S., & Freije, W. A. (2019). Maternal perinatal calorie restriction temporally regulates the hepatic autophagy and redox status in male rat. *Free Radical Biology & Medicine*, *130*, 592–600. 10.1016/j.freeradbiomed.2018.09.029 30248445 10.1016/j.freeradbiomed.2018.09.029 PMC8278542

[66] Liu, X., Wang, J., Gao, L., Jiao, Y., & Liu, C. (2018). Maternal protein restriction induces alterations in hepatic unfolded protein Response-Related molecules in adult rat offspring. *Frontiers in Endocrinology*, *9*, 676. 10.3389/fendo.2018.00676 30524373 10.3389/fendo.2018.00676 PMC6262354

[67] Honório, M., Martimiano, P., de Sa Braga Oliveira, A., Ferchaud-Roucher, V., Croyal, M., Aguesse, A., Grit, I., Ouguerram, K., de Lopes, S., Kaeffer, B., & Bolaños-Jiménez, F. (2017). Maternal protein restriction during gestation and lactation in the rat results in increased brain levels of kynurenine and kynurenic acid in their adult offspring. *Journal of Neurochemistry*, *140*(1), 68–81. 10.1111/jnc.13874 27778340 10.1111/jnc.13874

[68] Rosario, F. J., Jansson, N., Kanai, Y., Prasad, P. D., Powell, T. L., & Jansson, T. (2011). Maternal protein restriction in the rat inhibits placental insulin, mTOR, and STAT3 signaling and down-regulates placental amino acid transporters. *Endocrinology*, *152*(3), 1119–1129. 10.1210/en.2010-1153 21285325 10.1210/en.2010-1153 PMC3858644

[69] Gonzalez, P. N., Gasperowicz, M., Barbeito-Andrés, J., Klenin, N., Cross, J. C., & Hallgrímsson, B. (2016). Chronic protein restriction in mice impacts placental function and maternal body weight before fetal growth. *PloS One*, *11*(3), e0152227. 10.1371/journal.pone.0152227 27018791 10.1371/journal.pone.0152227 PMC4809512

[70] Reed, S. A., Ashley, R., Silver, G., Splaine, C., Jones, A. K., Pillai, S. M., Peterson, M. L., Zinn, S. A., & Govoni, K. E. (2022). Maternal nutrient restriction and over-feeding during gestation alter expression of key factors involved in placental development and vascularization. *Journal of Animal Science*, *100*(6), skac155. 10.1093/jas/skac155 35648126 10.1093/jas/skac155 PMC9159059

[71] Depierreux, D. M., Kieckbusch, J., Shreeve, N., Hawkes, D. A., Marsh, B., Blelloch, R., Sharkey, A., & Colucci, F. (2022). Beyond maternal tolerance: Education of uterine natural killer cells by maternal MHC drives fetal growth. *Frontiers in Immunology*, *13*, 808227. 10.3389/fimmu.2022.808227 35619712 10.3389/fimmu.2022.808227 PMC9127083

[72] Gong, M., Antony, S., Sakurai, R., Liu, J., Iacovino, M., & Rehan, V. K. (2016). Bone marrow mesenchymal stem cells of the intrauterine growth-restricted rat offspring exhibit enhanced adipogenic phenotype. International journal of obesity (2005), 40(11), 1768–1775. 10.1038/ijo.2016.157 10.1038/ijo.2016.157 PMC5113998 27599633

[73] Oreffo, R. O., Lashbrooke, B., Roach, H. I., Clarke, N. M., & Cooper, C. (2003). Maternal protein deficiency affects mesenchymal stem cell activity in the developing offspring. *Bone*, *33*(1), 100–107. 10.1016/s8756-3282(03)00166-2 12919704 10.1016/s8756-3282(03)00166-2

[74] Wang, C., Liu, J., Yan, Y., & Tan, Y. (2022). Role of Exosomes in Chronic Liver Disease Development and Their Potential Clinical Applications. Journal of immunology research, 2022, 1695802. 10.1155/2022/1695802 10.1155/2022/1695802 PMC9106457 35571570

[75] Ilarraza, R., Chao, D. V., Bodman, J. A. R., Chesley, A., Humble, A., Shaheen, F., Eitzen, G., & Lacy, P. (2023). Rac2 regulates immune complex-mediated granule polarization and exocytosis in neutrophils. *Journal of Leukocyte Biology*, *114*(2), 116–125. 10.1093/jleuko/qiad032 37017007 10.1093/jleuko/qiad032

[76] Valadi, H., Ekström, K., Bossios, A., Sjöstrand, M., Lee, J. J., & Lötvall, J. O. (2007). Exosome-mediated transfer of mRNAs and MicroRNAs is a novel mechanism of genetic exchange between cells. *Nature Cell Biology*, *9*(6), 654–659. 10.1038/ncb1596 17486113 10.1038/ncb1596

[77] Luo, X., Huang, B., Xu, P., Wang, H., Zhang, B., Lin, L., Liao, J., Hu, M., Liu, X., Huang, J., Fu, Y., Kilby, M. D., Kellems, R. E., Fan, X., Xia, Y., Baker, P. N., Qi, H., & Tong, C. (2025). The Placenta Regulates Intrauterine Fetal Growth via Exosomal PPARγ. Advanced science (Weinheim, Baden-Wurttemberg, Germany), 12(15), e2404983. 10.1002/advs.202404983 10.1002/advs.202404983 PMC12005745 39951006

[78] Nair, S., & Salomon, C. (2020). Extracellular vesicles as critical mediators of maternal-fetal communication during pregnancy and their potential role in maternal metabolism. *Placenta*, *98*, 60–68. 10.1016/j.placenta.2020.06.011 33039033 10.1016/j.placenta.2020.06.011

[79] Miranda, J., Paules, C., Nair, S., Lai, A., Palma, C., Scholz-Romero, K., Rice, G. E., Gratacos, E., Crispi, F., & Salomon, C. (2018). Placental exosomes profile in maternal and fetal circulation in intrauterine growth restriction - Liquid biopsies to monitoring fetal growth. *Placenta*, *64*, 34–43. 10.1016/j.placenta.2018.02.006 29626979 10.1016/j.placenta.2018.02.006

[80] Sánchez-Rubio, M., Abarzúa-Catalán, L., Del Valle, A., Méndez-Ruette, M., Salazar, N., Sigala, J., Sandoval, S., Godoy, M. I., Luarte, A., Monteiro, L. J., Romero, R., Choolani, M. A., Wyneken, Ú., Illanes, S. E., & Bátiz, L. F. (2024). Maternal stress during pregnancy alters Circulating small extracellular vesicles and enhances their targeting to the placenta and fetus. *Biological Research*, *57*(1), 70. 10.1186/s40659-024-00548-4 39342314 10.1186/s40659-024-00548-4 PMC11438166

